# Comparison between single-muscle evaluation and cross-sectional area muscle evaluation for predicting the prognosis in patients with oral squamous cell carcinoma: a retrospective cohort study

**DOI:** 10.3389/fonc.2024.1336284

**Published:** 2024-05-01

**Authors:** Hirotaka Takayama, Takuya Yoshimura, Hajime Suzuki, Yuka Hirano, Masahiro Tezuka, Takayuki Ishida, Kiyohide Ishihata, Marie Amitani, Haruka Amitani, Yasunori Nakamura, Yasushi Imamura, Akio Inui, Norifumi Nakamura

**Affiliations:** ^1^ Department of Oral and Maxillofacial Surgery, Kagoshima University Graduate School of Medical and Dental Sciences, Kagoshima, Japan; ^2^ Department of Community-Based Medicine, Kagoshima University Graduate School of Medical and Dental Sciences, Kagoshima, Japan; ^3^ Department of Psychosomatic Internal Medicine, Kagoshima University Graduate School of Medical and Dental Sciences, Kagoshima, Japan; ^4^ Department of Oral Surgery, Kagoshima Medical Center, National Hospital Organization, Kagoshima, Japan; ^5^ Department of Internal Medicine, Kagoshima Kouseiren Hospital, Kagoshima, Japan; ^6^ Pharmacological Department of Herbal Medicine, Kagoshima University Graduate School of Medical and Dental Sciences, Kagoshima, Japan

**Keywords:** oral cancer, sarcopenia, sliceOmatic^®^, Harrell’s concordance index, C-index

## Abstract

**Introduction:**

The most effective method of assessing sarcopenia has yet to be determined, whether by single muscle or by whole muscle segmentation. The purpose of this study was to compare the prognostic value of these two methods using computed tomography (CT) images in patients with oral squamous cell carcinoma (OSCC).

**Materials and methods:**

Sex- and age-adjusted Cox proportional hazards models were employed for each parameter of sarcopenia related to overall survival, disease-free survival, and disease-specific survival. Harrell’s concordance index was calculated for each model to assess discriminatory power.

**Results:**

In this study including 165 patients, a significant correlation was found between the CT-based assessment of individual muscles and their cross-sectional area. Single muscle assessments showed slightly higher discriminatory power in survival outcomes compared to whole muscle assessments, but the difference was not statistically significant, as indicated by overlapping confidence intervals for the C-index between assessments. To further validate our measurements, we classified patients into two groups based on intramuscular adipose tissue content (P-IMAC) of the spinous process muscle. Analysis showed that the higher the P-IMAC value, the poorer the survival outcome.

**Conclusion:**

Our findings indicate a slight advantage of single-muscle over whole-muscle assessment in prognostic evaluation, but the difference between the two methods is not conclusive. Both assessment methods provide valuable prognostic information for patients with OSCC, and further studies involving larger, independent cohorts are needed to clarify the potential advantage of one method over the other in the prognostic assessment of sarcopenia in OSCC.

## 1 Introduction

The prevalence of head and neck cancer is growing yearly, with oral squamous cell carcinoma (OSCC), the most frequent kind of oral cancer, having the highest mortality rate (1). In the United States, the total mortality rate for cancers of the oral cavity and pharynx increased by 0.5% per year from 2009 to 2018 (1). In 2020, there were approximately 53,260 new cases of this cancer, which is an increase of approximately 10,750 deaths compared to previous years (2). With advances in diagnostic and therapeutic modalities, 5-year survival rates for OSCC have improved markedly (3). An international retrospective study focused primarily on survival rates for OSCC patients treated with surgery alone or surgery and adjuvant radiation therapy (4). The results revealed that between 1990 and 2011, the 5-year survival rate for these patients increased from 59% to 70% (4).

Clinical symptoms associated with cancer progression in patients with OSCC can include limited mouth opening, tissue swelling, swollen lymph nodes, anorexia, and hygiene problems (5). Limited mouth opening, also known as trismus, can result from tumor growth or radiation therapy, leading to difficulties in eating, speaking, and maintaining oral hygiene (6). Tissue swelling and swollen lymph nodes may indicate disease progression and the spread of cancer (6). Advanced oral cancer is frequently linked to symptoms such as anorexia, loss of appetite, and consequent weight loss (6). It is known that patients with oral cancer are more likely to experience dysphagia due to the characteristics of the site of occurrence and are at high risk of deterioration of nutritional status and progression to frailty and sarcopenia (7). Sarcopenia is a condition in which muscle mass is reduced due to aging, malnutrition, and lack of physical activity, causing functional deterioration. Globally, especially in industrialized countries, the elderly population is growing (8–10), as is the number of elderly cancer patients. Elderly people and those with malnutrition, prolonged hospital stays, and reduced physical activity further promote sarcopenia (8–12). Sarcopenia itself is not a direct cause of decreased quality of life (QOL) in cancer patients. However, the underlying factors associated with sarcopenia, such as decreased physical function, fatigue, and treatment tolerance, can contribute to a decline in QOL among cancer patients. There is limited specific research on the direct relationship between sarcopenia and QOL in cancer patients. However, several studies have investigated the impact of muscle loss, physical function, and related factors on QOL in cancer patients (13, 14). The association between sarcopenia and poor postoperative prognosis has been described in various cancers, such as colorectal cancer and gastric cancer patients, and there are some reports that patients with preoperative sarcopenia have a poor postoperative prognosis (15–17). In the area of OSCC, the impact of sarcopenia on prognosis is becoming a focus of interest. The study by Lin et al. and the systematic review and meta-analysis by Graves et al. are pioneering works that evaluated the prognostic value of sarcopenia in OSCC (18, 19). Lin et al. demonstrated a significant relationship between reduced skeletal muscle mass and lower survival rates, suggesting the importance of sarcopenia as a prognostic factor in OSCC patients undergoing surgery (18). Similarly, Graves et al. highlighted the association between sarcopenia detected through radiographic assessment before treatment and poorer survival outcomes in patients with OSCC (19), supporting the findings of Lin et al.

Numerous techniques have been proposed to evaluate sarcopenia. To determine muscle mass or lean body mass, imaging modalities such as computed tomography (CT), magnetic resonance imaging (MRI), and dual-energy X-ray absorptiometry (DXA) have been employed. CT has gained popularity in recent times, especially among patients who opt for CT scans as part of their routine medical examination (20). Several studies have utilized CT scans to measure skeletal muscle mass by analyzing the cross-sectional area at the third lumbar vertebra (L3), which is highly correlated with total skeletal muscle mass (16). However, abdominal CT imaging is not a standard procedure for patients with squamous cell carcinoma of the head and neck, and lumbar region imaging is typically performed using ^18^F-fluorodeoxyglucose positron emission tomography/computed tomography (FDG PET/CT) for routine staging purposes (21–23).

To date, no definitive conclusion has been reached as to whether assessment by a single muscle or its whole cross-sectional area (CSA) is more appropriate in assessing sarcopenia. The psoas muscle index is often used to diagnose preoperative sarcopenia, but there is literature that requires muscle evaluation of the entire cross-section instead of a single muscle in the diagnosis of sarcopenia (20), while single-muscle evaluation has been related to poor prognosis in patients with OSCC (24, 25). Rollins KE et al. reported a correlation between the CSA of the psoas major muscle measured by CT and the CSA of the entire L3 slice for healthy subjects (26). However, they noted that this relationship was not conclusive and that single muscles should not be used as sentinels for whole-slice CSA because there was considerable variation in the measurements (26). On the other hand, it is also recommended that further research be conducted on the correlation between body composition analysis of individual skeletal muscle groups across L3 slices and clinical outcome measures, with the goal of assessing which muscle groups correlate best with the relevant clinical outcome measures (26).

Previous studies provide a basis for understanding the prognostic significance of sarcopenia in OSCC and advocate the assessment of sarcopenia in the prognostic evaluation of patients. Inspired by these findings, the present study examined whether the assessment of a single-muscle or the whole-muscle cross-sectional area is a more accurate predictor of prognosis in OSCC. Through this study, we sought to contribute new knowledge on the prognostic value of muscle assessment in OSCC, strengthen the current knowledge base, and advance our understanding of the relationship between muscle composition and cancer prognosis.

## 2 Materials and methods

### 2.1 Patients

**Figure 1:**
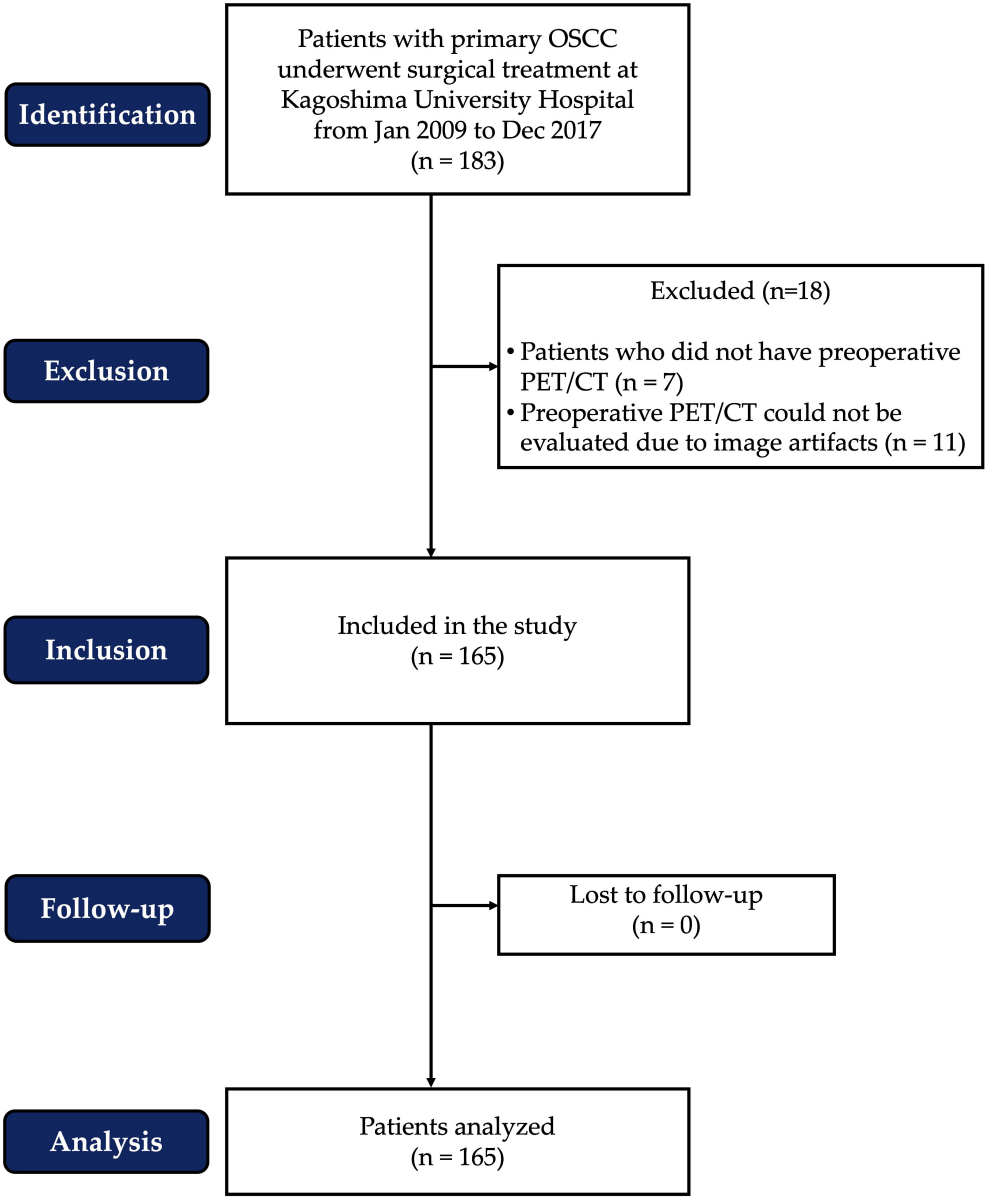
The patient flow chart according to STROBE standards.

Between January 2009 and December 2017, surgical treatment was performed on 183 patients with primary OSCC at Kagoshima University Hospital. However, some patients were excluded from the analysis due to the lack of preoperative FDG PET/CT imaging or artifacts in the patient’s CT images. A total of 165 patients were included in this retrospective cohort study. Gender, age, body mass index (BMI), comorbidities, tobacco/alcohol use, serum albumin concentration, tumor site, TN staging, tumor stage, treatment, survival rate, and duration of follow-up were among the clinicopathological data collected from all patients. The patient flow chart, according to STROBE standards, is presented in **Figure 1**.

We utilized the TNM classification system of the International Union Against Cancer, specifically the 7th edition of 2010 (27), to determine the clinical and pathological stages of each patient. The 7th edition was chosen for data collection because it was the most recent edition available at the time and allowed for consistency in patient classification. Based on clinical and imaging findings, a cervical dissection was planned for each patient. Patients with confirmed lymph node metastases [cN(+)] or requiring reconstructive surgery underwent simultaneous neck dissection with resection of the primary tumor. Radical neck dissection involving levels I-V on the affected side was performed if lymph node metastases were detected prior to treatment. During cervical dissection, the sternocleidomastoid muscle was always excised, but the accessory nerves and the internal jugular vein were preserved if they were not directly involved in the metastatic lymph nodes.

For the purpose of monitoring the patient’s postoperative recovery and to identify signs of recurrence or complications, routine clinical examinations were performed by the surgeon after surgery. The frequency varied over time: once or twice a month in the first year, once a month in the second year, and every three to six months in the third to fifth years. Contrast-enhanced CT and ultrasound were performed at regular intervals after surgery. Imaging was performed at 1, 3, 6, 12, 18, and 24 months postoperatively and annually for the following three years. If cervical lymph node metastases were detected during the surveillance period, immediate cervical dissection and histopathology were performed to further investigate and determine the extent of metastases.

### 2.2 Ethics and informed consent statement

This retrospective cohort study adhered to the principles of the Helsinki Declaration and was approved by the Institutional Ethics Committee of Kagoshima University (permission No. 160319). Due to the nature of the investigation, patient consent was not obtained. Instead, an opt-out approach was implemented, and a notification was posted on the Kagoshima University Hospital website to obtain authorization for this study.

### 2.3 ^18^F FDG-PET/CT scanning

The detection of metastasis, including metastatic lymph nodes, using 18F FDG-PET/CT has been established. The Discovery PET/CT 600 Motion and Discovery PET/CT 610 Motion (GE Healthcare) were employed to obtain images for this study. Patients were administered 18F FDG at a dose of 3.7 MBq/kg body weight after fasting for 6 hours before the 18F FDG-PET/CT examinations. Whole-body scanning was performed one hour after the administration of 18F FDG, followed by local imaging two hours later.

### 2.4 Image analysis

**Figure 2:**
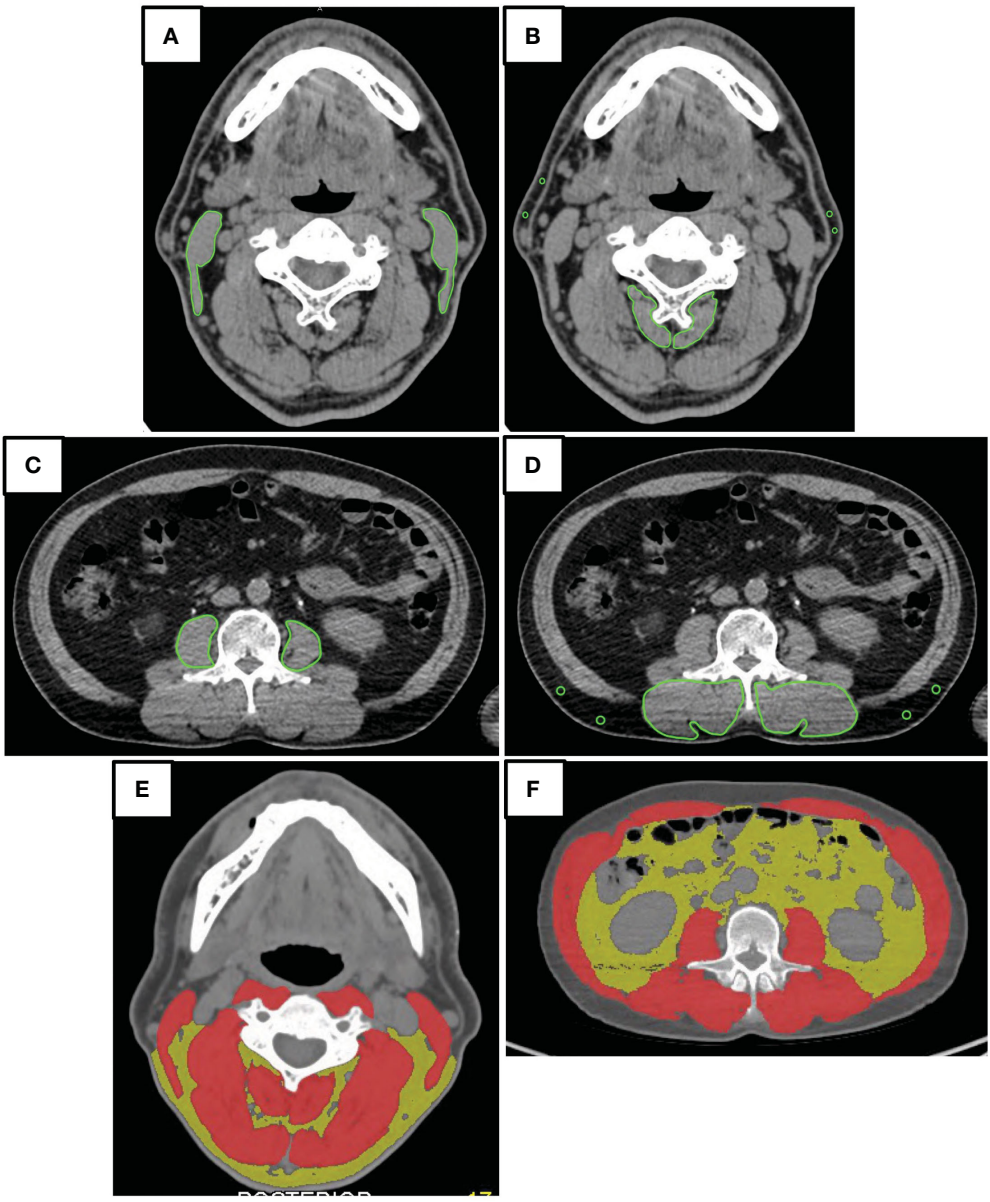
Image analysis. The illustrative outcomes from a comprehensive analysis of muscular and adipose tissues using imaging techniques. **(A, C)** Tracing of Muscular Contours for Quantitative Assessment: The precise delineation of the multifidus and sternocleidomastoid muscles facilitated the calculation of the Spinal Cord Muscle Index (SCMI) and the Psoas Muscle Index (PMI). By normalizing the cross-sectional areas of muscle (in cm²) against the height of the subjects, indices were derived in units of cm²/m². This normalization process permitted a uniform comparison among subjects, regardless of their height variations. **(B, D)** The Evaluation of Muscle Quality through Adipose Tissue Ratios: Through the calculation of the Posterior Intermuscular Adipose Tissue Composition (P-IMAC) and the Intermuscular Adipose Composition (IMAC), an assessment of muscle quality was achieved. This assessment required dividing the computed tomography (CT) values for the bilateral multifidus muscle at the cervical level 3 (C3) and lumbar level 3 (L3) by the CT attenuation values of subcutaneous adipose tissue. The resulting ratios served as metrics for analyzing the infiltration of fat into the muscle, offering insights into muscle quality. **(E, F)** The Quantification of Cross-Sectional Areas at Specific Vertebral Levels: These panels provided a visual and quantitative analysis of the cross-sectional areas at the C3 and L3 levels. An emphasis was placed on differentiating between muscular and adipose tissues, with muscular tissues highlighted in red and adipose tissues depicted in yellow. This color differentiation facilitated a clear visual distinction, which in turn supported the quantitative analysis by clearly delineating the areas of interest for accurate measurement.

To identify the presence of systemic metastasis of squamous cell carcinoma, a single FDG PET/CT scan was performed within two weeks before surgery. The CT component of the FDG PET/CT images was utilized for image analysis. A single measurer, who was not involved in the treatment, performed the analysis based on a previous study (28). The CT values for skeletal muscle were defined as areas of -29 to 150 HU, while subcutaneous adipose tissue areas were defined as areas of -150 to -50 HU. The multifidus and sternocleidomastoid muscles were evaluated separately by tracing their contours in OsiriX v.4.0 (Pixmeo SARL, Geneva, Switzerland). The sternocleidomastoid muscle mass index (SCMI) and psoas muscle mass index (PMI) were calculated by normalizing cross-sectional areas to height (cm^2^/m^2^) (25). The region of interest (ROI) for the multifidus muscle was used to determine the processus spinosus muscle-intramuscular adipose tissue content (P-IMAC) and intramuscular adipose tissue content (IMAC) by dividing the ROI of the multifidus muscle (in Hounsfield units) by the ROI of subcutaneous adipose tissue (in Hounsfield units) (25). The cross-sectional areas (CSA) (in cm^2^) of skeletal muscle in the third cervical vertebra (C3) and the third lumbar vertebrae (L3) region, as well as CT values (in Hounsfield units), were evaluated using sliceOmatic^®^ v.5.0 (TomoVision, Magog, Canada). The areas of muscle and adipose tissue were traced semiautomatically. Adipose tissue of the same cross-section was measured, and the average CT value was used as the CT value of adipose tissue. Cross-sectional areas were normalized by height (in cm^2^) and defined as the skeletal muscle index (SMI) and C3-SMI, respectively. The cross-sectional area intramuscular adipose tissue content (CSA IMAC) and the cross-sectional area intramuscular adipose tissue content at the level of the third cervical vertebra (C3-CSA IMAC) were defined by dividing SMI and C3-SMI by the CT value of adipose tissue, respectively. A summary of the parameters is presented in **Table 1**. **Figure 2** is an analysis of muscle and adipose tissue by image, highlighting the delineation and quantitative evaluation of the multifidus and sternocleidomastoid muscles. This analysis facilitated the calculation of the SCMI and PMI by normalizing the muscle cross-sectional area to the height of the subject, allowing uniform comparisons. In addition, the ratio of CT values of muscle to subcutaneous adipose tissue was calculated to assess muscle quality and provide insight into fat infiltration. This visualization enabled the distinction between muscle and adipose tissue and the accurate quantification of cross-sectional area at a given vertebral level.

**Table 1:** Indicators for assessing sarcopenia in OSCC: single muscle and cross-sectional area analysis.

The scope of the muscle analysis	Evaluation site	Name
Single muscle	Lumbar	PMI
		IMAC
	Cervical	SCMI
		P-IMAC
CSA-muscle	Lumbar	SMI
		CSA IMAC
	Cervical	C3-SMI
		C3-CSA IMAC

OSCC, oral squamous cell carcinoma; CSA, cross-sectional area; PMI, psoas muscle mass index; IMAC, intramuscular adipose tissue content; SCMI, sternocleidomastoid muscle mass index; P-IMAC, processus spinosus muscle—intramuscular adipose tissue content; SMI, skeletal muscle index; CSA IMAC, cross-sectional area intramuscular adipose tissue content; C3-SMI, skeletal muscle index in the third cervical vertebra; C3-CSA IMAC, cross-sectional area intramuscular adipose tissue content in the third cervical vertebra.

### 2.5 Parameter analysis

To assess the relationships between single muscle and CSA muscle evaluation, we investigated correlations across several key metrics: PMI and SMI, IMAC and CSA IMAC, SCMI and C3-SMI, as well as P-IMAC and C3-CSA IMAC. To quantify these associations, Pearson’s correlation analyses were performed, followed by simple linear regression analysis to further elucidate the strength and nature of these relationships. Sex-, age, and stage-adjusted Cox proportional hazards models were created for each parameter for overall survival (OS), disease-free survival (DFS), and disease-specific survival (DSS). After every survey estimation, Harrell’s concordance index (C-index) of each model was evaluated for linear combinations of coefficients (29).

### 2.6 Statistical analysis

Statistical significance was determined by P values minor to 0.05. The Youden index was employed to determine the appropriate cutoff values for variable estimates. The Kaplan−Meier method was utilized to analyze the survival curves, and the log-rank test was performed to compare the survival curves between the two groups. Stata version 16 (StataCorp LLC, College Station, TX, USA) and GraphPad Prism version 9.5.0 for MacOS (GraphPad Software, San Diego, CA, USA) were used for all statistical analyses.

## 3 Results

### 3.1 Sample characteristics

**Figure 3:**
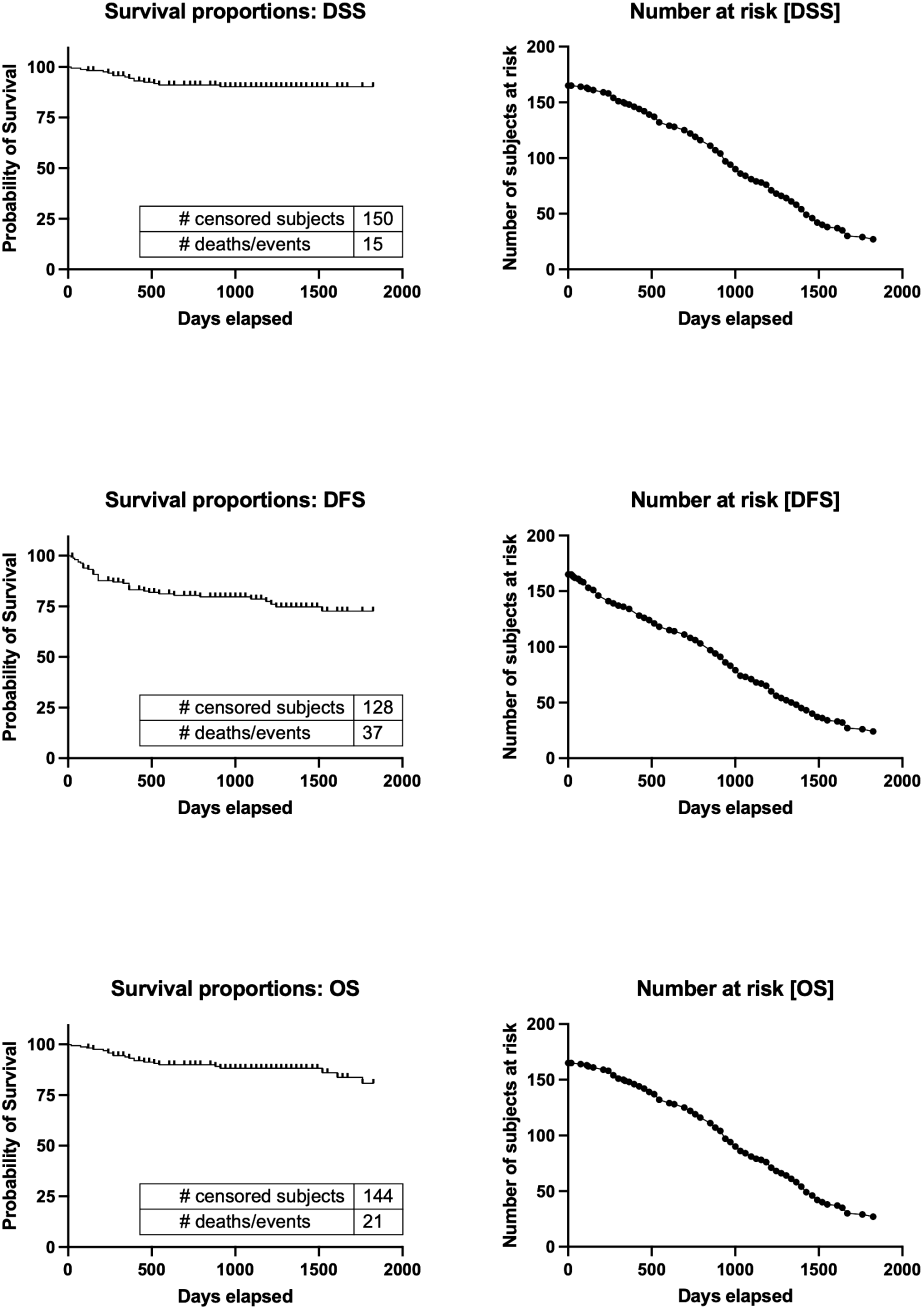
Kaplan−Meier curves and the number of subjects at risk of overall DSS, DFS, and OS. Kaplan-Meier survival curves for Disease-Specific Survival (DSS), Disease-Free Survival (DFS), and Overall Survival (OS), alongside the number of subjects at risk over a timeline extending to 2000 days. It illustrates survival probabilities from diagnosis, detailing the chance of avoiding disease-specific death, recurrence, and any cause of death, respectively. Through censoring points and event occurrences, the figure reveals trends in survival probabilities across different periods. Additionally, it highlights the changing cohort size at risk at key intervals, providing a dynamic view of the study population’s survival over time. This visualization offers a succinct overview of survival outcomes and temporal disease progression dynamics. DSS, disease-specific survival; DFS, disease-free survival; OS, overall survival.

**Table 2** presents an overview of the patient characteristics. In summary, males accounted for over half of the patients (64%), and the average age was 68 years. The patients had a normal body mass index (BMI) on average. The primary tumors invaded the oral mucosa in various locations, including the tongue, gingiva, oral floor, buccal region, palate, and lip. At the time of diagnosis, 60% of patients had advanced disease (TNM stages III and IV). The 5-year disease-specific survival rate for all patients was 88.1%, the 5-year disease-free survival rate was 69.9%, and the 5-year overall survival rate was 79.7%. The median duration of follow-up was 1060 days. The Kaplan−Meier curves and the number of subjects at risk of overall DSS, DFS, and OS, alongside graphical representations of the number of subjects at risk over time was shown in **Figure 3**. These results provided a comprehensive overview of survival outcomes and subject risk, employing Kaplan-Meier estimations to elucidate the temporal dynamics of survival and disease progression among the samples.

**Table 2:** Sample characteristics. (A) Demographic and clinical characteristics of the study population (n=165).

Characteristics	
Sex	
Male	108 (65.5%)
Female	57 (34.5%)
Age (years)	
Male	67.16 ± 10.59
Female	68.33 ± 14.20
BMI (kg/m^2^)	
Male	23.27 ± 3.30
Female	22.21 ± 3.54
Comorbidities (yes)	101 (61.2%)
Tobacco/Alcohol Use	67.7%
Albumin (g/dL)	
Male	4.272 ± 0.418
Female	4.444 ± 0.408
Clinical characteristics	
Tumor Location	
Tongue	74 (44.8%)
Gingival	58 (35.2%)
Oral floor	16 (9.7%)
Buccal	10 (6.1%)
Palate	6 (3.6%)
Lip	1 (0.6%)
Classification	
T1	30 (18.2%)
T2	71 (43.0%)
T3	33 (20.0%)
T4	23 (13.9%)
Nodal Involvement	
N0	92 (55.8%)
N1	32 (19.4%)
N2	38 (23.0%)
N3	3 (1.8%)
Stage	
I	25 (15.2%)
II	37 (22.4%)
III	41 (24.8%)
IV	54 (32.7%)
Treatment	
Surgery only	103 (62.4%)
Surgery with RT/CT	62 (37.6%)
Survival Outcomes	
5-Year Disease-Specific Survival	88.1% (145 out of 165 subjects)
5-Year Disease-Free Survival	69.9% (115 out of 165 subjects)
5-Year Overall Survival	79.7% (131 out of 165 subjects)
Follow-Up Duration (days): Median [Q1-Q3]	1060 [695-1490]

**Table 2 (B):** Image and preoperative parameters.

	Male (n=108)	Female (n=57)
Preoperative PMI (cm^2^/m^2^)	6.437 ± 1.787	4.561 ± 1.270
Preoperative IMAC	-0.4085 ± 0.1196	-0.2537 ± 0.2275
Preoperative SCMI (cm^2^/m^2^)	2.420 ± 1.543	1.737 ± 0.5407
Preoperative P-IMAC	-1.140 ± 8.409	-0.2531 ± 0.1660
Preoperative SMI (cm^2^/m^2^)	46.64 ± 7.403	38.03 ± 5.154
Preoperative CSA IMAC	1.062 ± 1.143	0.9602 ± 1.524
Preoperative C3-SMI (cm^2^/m^2^)	14.53 ± 2.439	11.92 ± 1.982
Preoperative C3-CSA IMAC	0.4713 ± 0.098	0.4265 ± 0.1547

Continuous data are presented as means ± standard deviation.

BMI, body mass index; Q1, 25% quantile; Q3, 75% quantile; PMI, psoas muscle mass index; IMAC, intramuscular adipose tissue content; SCMI, sternocleidomastoid muscle mass index; P-IMAC, processus spinosus muscle—intramuscular adipose tissue content; SMI, skeletal muscle index; CSA IMAC, cross-sectional area intramuscular adipose tissue content; C3-SMI, skeletal muscle index at the level of the third cervical vertebra; C3-CSA IMAC, cross-sectional area intramuscular adipose tissue content at the level of the third cervical vertebra.

### 3.2 The correlations between the single muscle evaluation and cross-sectional area muscle evaluation

**Figure 4:**
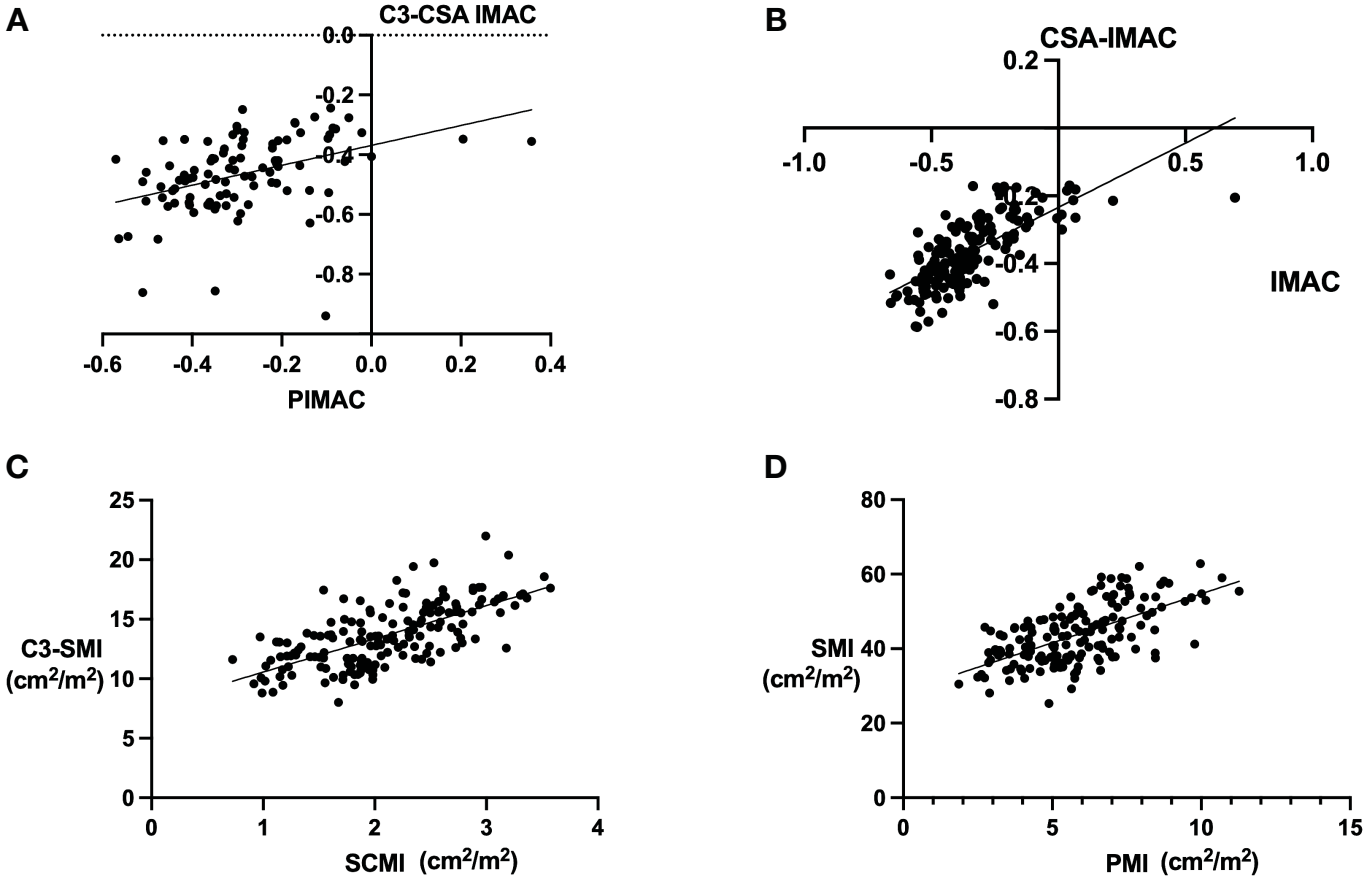
Correlations between the single muscle evaluation and cross-sectional area (CSA) muscles. The image shows correlations between various muscular and adipose tissue metrics as determined by Pearson’s correlation coefficient. Panel **(A)** displays the relationship between cross-sectional area (CSA) at the cervical level 3 (C3) intermuscular adipose composition (IMAC) and posterior intermuscular adipose tissue composition (P-IMAC), revealing a Pearson’s r value of 0.4082 (P<0.0001). Panel **(B)** demonstrates the correlation between CSA-IMAC and IMAC, with a Pearson’s r of 0.7136 (P<0.0001). Panel **(C)** presents the association between C3 spinal muscle index (SMI) and spinal cord muscle index (SCMI), indicating a Pearson’s r of 0.6702 (P<0.0001). Lastly, Panel **(D)** shows the correlation between psoas muscle index (PMI) and SMI, with a Pearson’s r of 0.6210 (P<0.0001). These analyses underscore significant associations between key indices of muscle and adipose tissue composition, highlighting the interconnectedness of these metrics in the context of patient evaluation and disease prognosis.

Significant correlations were identified in several muscle and adipose tissue indices in our study. The relationship between C3-CSA IMAC and P-IMAC was demonstrated with a Pearson correlation coefficient (r) of 0.4082 (95% CI: 0.2273 - 0.5919; P<0.0001; **Figure 4A**), and a regression slope of 0.3334 (95% CI: 0.1815 – 0.4852), yielding a t-value of 19.0 at 95 degrees of freedom (P<0.0001) and an explained variance of 16.67% (r² = 0.1667). Similarly, CSA-IMAC and IMAC showed a Pearson’s r of 0.7136 (95% CI: 0.6295 - 0.7812; P<0.0001; **Figure 4B**), with a regression slope of 0.3794 (95% CI: 0.3218 – 0.4370), t-value of 169.1 at 163 degrees of freedom (P<0.0001), and an r² of 0.5093. The correlation between C3-SMI and SCMI was also strong, with a Pearson’s r of 0.6702 (95% CI: 0.5761 - 0.7468; P<0.0001; **Figure 4C**), regression slope of 2.792 (95% CI: 2.312 – 3.272), t-value of 132.1 at 162 degrees of freedom (P<0.0001), and an r² of 0.4492. Finally, PMI and SMI correlation yielded a Pearson’s r of 0.6210 (95% CI: 0.5173 - 0.7067; P<0.0001; **Figure 4D**), with a slope of 2.632 (95% CI: 2.118 – 3.416), t-value of 102.3 at 163 degrees of freedom (P<0.0001), and an r² of 0.3857. These analyses underscore significant associations between key indices of muscle and adipose tissue composition, highlighting the interconnectedness of these metrics in the context of patient evaluation and disease prognosis.

### 3.3 Evaluation and comparison of Cox proportional hazards models of survival time analysis with the C-index

**Figure 5:**
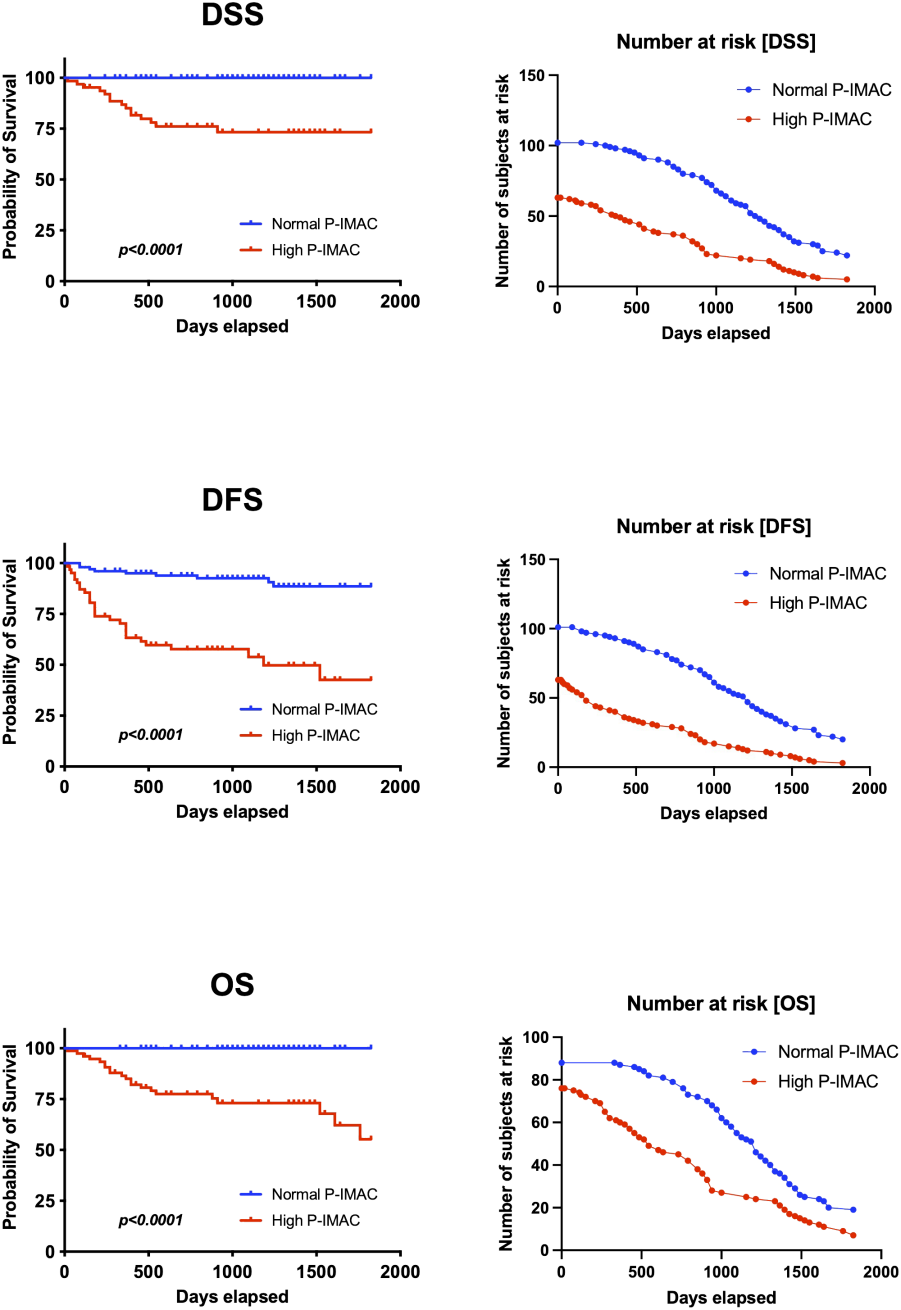
Survival analysis. P-IMAC quantifies the intramuscular adipose tissue content within the processus spinosus muscle. The analysis distinctly showed that subjects classified within the high P-IMAC group exhibit significantly lower survival rates across all measured endpoints when compared to those in the normal P-IMAC group. P-IMAC, processus spinosus muscle—intramuscular adipose tissue content; OS, overall survival; DFS, disease-free survival; DSS, disease-specific survival.

**Table 3** displays the discriminating power of P-IMAC and SCMI in predicting OS, DFS, and DSS prognosis. P-IMAC demonstrated the strongest discriminating power for predicting OS, with a C-index of 0.799 (95% CI 0.731-0.866), followed by SCMI with a C-index of 0.790 (95% CI 0.689-0.892). Similarly, P-IMAC had the strongest discriminating power for predicting DFS, with a C-index of 0.742 (95% CI 0.653-0.831), followed by SCMI with a C-index of 0.647 (95% CI 0.549-0.746). In predicting DSS, P-IMAC showed the strongest discriminating power with a C-index of 0.825 (95% CI 0.753-0.896), followed by SCMI with a C-index of 0.824 (95% CI 0.722-0.927). Patients were divided into two groups based on their P-IMAC levels: those with “normal P-IMAC” below the cutoff values and those with “high P-IMAC” above the cutoff values. High P-IMAC patients had significantly poorer survival rates in DSS, DFS, and OS than normal P-IMAC patients (P<0.0001; **Figure 5**). P-IMAC cutoff values for males and females were as follows: DSS: -0.3036 (AUC=0.86; sensitivity, 100%; specificity, 68%) and -0.2345 (AUC=0.67; sensitivity, 100%; specificity, 68%); DFS: -0.3036 (AUC=0.75; sensitivity, 72.2%; specificity, 70%) and -0.2345 (AUC=0.79; sensitivity, 79.0%; specificity, 79.0%); OS: -0.3393 (AUC=0.82; sensitivity, 100%; specificity, 58.5%) and -0.2345 (AUC=0.67; sensitivity, 100%; specificity, 68%).

**Table 3:** Harrell’s concordance index (C-index) in sex-, age, and stage-adjusted Cox proportional regression analysis.

			5-Year OS		5-Year DFS		5-Year DSS	
			C-index	95%CI	C-index	95%CI	C-index	95%CI
Single muscle	Lumbar	PMI	0.679	0.573-0.786	0.629	0.530-0.728	0.682	0.546-0.818
		IMAC	0.670	0.560-0.780	0.639	0.540-0.738	0.647	0.511-0.783
	Cervical	SCMI	0.790	0.689-0.892	0.647	0.549-0.746	0.824	0.722-0.927
		P-IMAC	0.799	0.731-0.866	0.742	0.653-0.831	0.825	0.753-0.896
CSA-muscle	Lumbar	SMI	0.676	0.564-0.788	0.622	0.523-0.721	0.644	0.507-0.781
		CSA IMAC	0.667	0.555-0.779	0.625	0.526-0.724	0.650	0.510-0.790
	Cervical	C3-SMI	0.680	0.568-0.792	0.624	0.524-0.725	0.649	0.510-0.789
		C3-CSA IMAC	0.677	0.575-0.779	0.624	0.525-0.723	0.654	0.542-0.767

CSA, cross-sectional area; PMI, psoas muscle mass index; IMAC, intramuscular adipose tissue content; SCMI, sternocleidomastoid muscle mass index; P-IMAC, processus spinosus muscle—intramuscular adipose tissue content; SMI, skeletal muscle index; CSA IMAC, cross-sectional area intramuscular adipose tissue content; C3-SMI, skeletal muscle index at the level of the third cervical vertebra; C3-CSA IMAC, cross-sectional area intramuscular adipose tissue content at the level of the third cervical vertebra; OS, overall survival; DFS, disease-free survival; DSS, disease-specific survival; C-index, Harrell’s concordance index; CI, confidence interval.

## 4 Discussion

This study retrospectively evaluated the prognostic value of single muscle assessment compared to whole cross-sectional area in patients with OSCC, and is the first attempt to determine which assessment method more accurately predicts prognosis in OSCC patients. Our findings fill an important gap in oncology research and introduce a new perspective on prognostic assessment, which may have a significant impact on patient management and treatment planning. The focus of previous studies has been on the relationship between whole muscle assessment and single muscle assessment (30, 31). Our study suggests that single muscle evaluation using cervical muscle CT scans is the most accurate predictor of 5-year overall survival, disease-free survival, and disease-specific survival in OSCC patients. In a previous study, we found that OSCC patients with low preoperative muscle mass (indicated by a low PMI) and high preoperative muscle volume (indicated by a high IMAC) had significantly lower disease specificity than the control group (24). It has also been suggested that SCMI and P-IMAC, which are C3-level muscle evaluations, may be superior to L3-level muscle evaluations among single muscles (25). The present study supports these previous studies and further indicates that the evaluation of a single neck muscle may be more useful than the evaluation of the CSA of the neck or lumbar in predicting a patient’s prognosis.

Sarcopenia is significantly influenced by molecular mechanisms, particularly in the context of cancer (32). Chronic systemic inflammation, marked by elevated levels of cytokines such as TNF-α and IL-6 (33), plays a pivotal role in muscle degradation. The ubiquitin-proteasome system, with key enzymes like Atrogin-1 and MuRF1, accelerates muscle protein breakdown, contributing to sarcopenia (34). Additionally, myostatin and activins inhibit muscle growth through the Smad 2/3 signaling pathway (35). Insulin resistance and mitochondrial dysfunction further exacerbate muscle loss by impairing energy production and increasing oxidative stress (36). Hormonal fluctuations, especially in testosterone and growth hormone, also contribute to sarcopenia’s development (37). Our findings, particularly the correlation between higher P-IMAC and poorer survival outcomes, align with the complex interplay of sarcopenia’s molecular mechanisms in cancer prognosis. Our study underscores the pivotal role of sarcopenia, propelled by multifaceted molecular dynamics, as a significant prognostic factor in OSCC, highlighting the potential of single-muscle CT evaluations in refining prognostic assessments and tailoring patient care strategies.

Treatments for patients with OSCC, such as surgery, radiation therapy, and chemotherapy, can significantly affect a patient’s ability to eat and swallow. In patients with OSCC, there is a significant relationship between low food intake and sarcopenia. Pain, mouth sores, difficulty chewing and swallowing, and changes in taste and appetite are common side effects of OSCC treatment (6). Dysphagia can make it difficult to consume adequate food and, if not managed appropriately, can lead to reduced food intake, malnutrition, and muscle wasting, such as sarcopenia. Nutritional deficiencies impair muscle protein synthesis, increase muscle breakdown, and accelerate the development of sarcopenia. In this context, past literature emphasizes the importance of optimal protein intake for preventing and managing sarcopenia, especially in the elderly (38–41). Exercise interventions, especially resistance training, have shown promising results in combating sarcopenia by increasing muscle mass, strength, and function (42). In addition, nutritional strategies focused on adequate protein intake and potentially beneficial supplements such as vitamin D, calcium, and branched-chain amino acids (BCAAs) provide another important avenue for sarcopenia management (41, 43). On the other hand, no studies have yet been conducted on OSCC patients, and further research is needed to prove the usefulness of these strategies in OSCC patients. Our study highlights the predictive value of a single muscle assessment for OSCC patient’s prognosis and integration of sarcopenia management into patient care, emphasizes the need for clinical trials to evaluate the effectiveness of exercise and nutrition interventions, and further research on their optimal timing, intensity, and customization to OSCC treatment strategies will provide important insights into the effective integration of sarcopenia management.

It is still a matter of debate as to which muscles best reflect systemic sarcopenia. Single muscle assessment is very simple and convenient (44), whereas total skeletal muscle mass assessment using automated total muscle segmentation on CT images is not very versatile in actual clinical practice in general clinics. However, since both muscle and intramuscular adipose tissue were measured in the single muscle assessment, the single muscle does not always reflect the actual muscle mass (45). Thus, expert panels have suggested that a single sentinel muscle is not recommended for diagnosing sarcopenia (46–48), as it is difficult to claim that one muscle is representative (20). Yoon et al. reported that cervical muscle strength assessment does not adequately reflect whole-body sarcopenia (49), while Bril et al. concluded that CSA at C3 can provide a good estimate of skeletal muscle mass (SMM) in patients with head and neck cancer, without the need for additional diagnostic procedures and with minimal effort and with considerable accuracy at present (30). However, in line with the study on Japanese patients with OSCC (31), they acknowledge that estimation of CSA at L3 based on CSA at C3 is not ideal and may overestimate patients with low SMM (30) Bril et al. also concluded that estimation of CSA is probably not sufficient as the most accurate estimator of a patient’s total SMM because the problem that a single muscle may not reflect the whole-body SMM, as concluded by Baracos et al. (20), probably applies to CSA on a single CT slice representing whole-body SMM (30). In a previous study, we suggested that assessing both PMI and IMAC (i.e., evaluating different muscles in combination) could serve as a substitute for evaluating muscle strength and function and could be the most suitable parameter for evaluating preoperative sarcopenia (24). Our study aimed to compare the quantification of skeletal muscle mass using both single muscle evaluation and CSA in CT images. Our findings indicate that single muscle evaluation had superior discrimination with the C-index compared to CSA. These results offer valuable insights into the ongoing debate about the usefulness of single-muscle versus CSA assessments.

There are several limitations to this study. First, sliceOmatic^®^ is designed by semiautomatically identifying a range of CT values; however, the design also includes other adjacent muscles and other tissues with the same CT value. Ultimately, the measurer must identify and design the tissues, which may lead to measurement errors. Second, because this was a retrospective study in a single cohort, patient selection bias could not be eliminated, and the generalizability of the findings may be limited. Because a control group of healthy subjects who were not head and neck cancer patients was not measured, the quality and quantity of each muscle could not be compared to a healthy control group. Recognizing the importance of validation, we acknowledge that cross-validation with another cohort with identical parameters and cutoff values is essential to confirm the applicability and robustness of these results. Future studies will incorporate independent cohorts to address this limitation, which will help establish the reliability and broad applicability of our findings in diverse patient populations. Third, because the measurements were performed by a single measurer, reliability, and validity have not been verified. Therefore, it is necessary to conduct measurements by multiple measures in the future to verify errors and confirm reproducibility. Fourth, the cutoff values for PMI and IMAC, as well as SCMI and P-IMAC, were established about the methods used in our previous studies (24, 25). The European Working Group on Sarcopenia in Older People (EWGSOP2) guidelines offer cutoff points for simple and specific measurements (50). However, importantly, these values were derived from primarily Caucasian populations and may not be directly applicable to Asian populations due to differences in body size, lifestyle, and ethnicity (51). Therefore, future prospective longitudinal intervention studies will be necessary to validate the proposed cutoff values. Lastly, the omission of a comprehensive assessment of adverse events related to oncologic treatment using established standard terminology criteria for adverse events (CTCAE). Furthermore, the Edmonton Symptom Assessment System (ESAS) subjective global rating scale, which comprehensively assesses patient symptoms and their severity, was not employed. The exclusion of these assessments precludes a detailed understanding of treatment-related side effects and their impact on patient quality of life, which is crucial to a holistic oncologic care approach. Future studies will incorporate these methodologies to provide a more comprehensive analysis of oncologic treatment outcomes and a better understanding of the balance between treatment efficacy and management of side effects. Incorporation of these methodologies will allow treatment to be tailored to minimize adverse effects on patient well-being and enhance overall treatment strategies, which is expected to contribute significantly to optimizing patient care.

Although the above limitations must be considered, this study suggests that a focus on single-strand assessment with CT imaging may provide better prognostic information for patients with oral squamous cell carcinoma. Incorporating this approach into clinical practice could enhance treatment decision-making and patient management strategies. However, individual clinical judgment must always be made, considering the characteristics of each patient and referring to other relevant evidence and guidelines.

## 5 Conclusions

Our study indicates that while CT imaging-based assessment of single muscles may have slight differences in prognostic predictions for OSCC patients compared to the evaluation of entire muscles through semiautomated segmentation, the results do not conclusively establish superior reliability. The overlapping confidence intervals for the C-index among the markers suggest that both methods provide comparably valuable prognostic information, while the simplicity and direct clinical applicability of single-muscle assessment might offer practical advantages. Given these findings, further prospective studies are essential to substantiate the clinical relevance and potentially refine the prognostic utility of single muscle assessment in this patient population.

## Data availability statement

The raw data supporting the conclusions of this article will be made available by the authors, without undue reservation.

## Ethics statement

The studies involving humans were approved by Institutional Ethics Committee of Kagoshima University (permission No. 160319). The studies were conducted in accordance with the local legislation and institutional requirements. Written informed consent for participation was not required from the participants or the participants’ legal guardians/next of kin because due to the nature of the investigation, patient consent was not obtained. Instead, an opt-out approach was implemented, and a notification was posted on the Kagoshima University Hospital website to obtain authorization for this study.

## Author contributions

HT: Investigation, Writing – original draft. TY: Conceptualization, Formal Analysis, Funding acquisition, Investigation, Methodology, Writing – review & editing. HS: Conceptualization, Formal Analysis, Funding acquisition, Writing – original draft, Writing – review & editing. YH: Investigation, Writing – review & editing. MT: Validation, Writing – review & editing. TI: Data curation, Software, Writing – review & editing. KI: Data curation, Validation, Writing – review & editing. MA: Writing – review & editing. HA: Writing – review & editing. YN: Supervision, Writing – review & editing. YI: Supervision, Writing – review & editing. AI: Conceptualization, Writing – review & editing. NN: Conceptualization, Project administration, Writing – review & editing.
